# Lead-associated Superior Vena Cava Syndrome

**DOI:** 10.19102/icrm.2021.120404

**Published:** 2021-04-15

**Authors:** Andrew H. Locke, David J. Shim, Jessica Burr, Tyler Mehegan, Kelsey Murphy, André D’Avila, Marc L. Schermerhorn, Peter Zimetbaum

**Affiliations:** ^1^Harvard-Thorndike Electrophysiology Institute, Beth Israel Deaconess Medical Center, Harvard Medical School, Boston, MA, USA; ^2^Boston Scientific, Natick, MA, USA; ^3^Department of Surgery, Division of Vascular and Endovascular Surgery, Beth Israel Deaconess Medical Center, Harvard Medical School, Boston, MA, USA; ^4^Harvard-Thorndike Electrophysiology Institute, Beth Israel Deaconess Medical Center, Harvard Medical School, Boston, MA, USA

**Keywords:** Anticoagulation, cardiovascular implantable electronic device, device extraction, superior vena cava syndrome, venoplasty

## Abstract

Superior vena cava (SVC) syndrome is a rare complication associated with transvenous cardiac implantable electronic devices that may present with a variety of manifestations. Various strategies such as transvenous lead extraction, anticoagulation, venoplasty, and stenting have been used to treat this condition, but the optimal management protocols have yet to be defined. Subcutaneous implantable cardioverter-defibrillator (ICD) (S-ICD) therapy can be an alternative option to a transvenous system for those who require future ICD surveillance. We present a case of lead-associated SVC syndrome where thoracic venous congestion due to SVC obstruction influenced preimplant S-ICD QRS vector screening. Following treatment of venous obstruction, QRS amplitude may change and patients who were not initially S-ICD candidates may later become eligible.

## Introduction

Superior vena cava (SVC) syndrome is a rare complication associated with transvenous cardiac implantable electronic devices that may present with a variety of manifestations, including facial plethora as well as more atypical symptoms such as subcutaneous thoracic congestion and ecchymoses from the recruitment of collateral circulation.^1^ The earliest case reports of SVC syndrome date back to the 18^th^ century and describe infectious etiologies such as tuberculous lymphadenitis and syphilitic aortitis causing external compression of the SVC.^2^ From the late 18^th^ century onward, malignancies—specifically lung cancer and non-Hodgkin’s lymphoma—are the most common etiologies of SVC syndrome. More recently, due to improvements in malignancy therapy, benign causes have become increasingly more prevalent, accounting for up to 40% of cases of SVC syndrome.^3^

Lead-associated SVC syndrome is an important cause of benign SVC syndrome. Each year in the United States, approximately 300,000 pacemakers and 100,000 implantable cardioverter-defibrillators (ICDs) are implanted. In studies evaluating venous vasculature in asymptomatic patients with transvenous leads, 31% to 64% have some degree of SVC obstruction.^4^ Despite that 400,000 device implants are performed annually in the United States every year, less than 1% develop lead-associated SVC syndrome. Multiple risk factors for this condition have been identified, but none have proven highly predictive of the development of lead-associated SVC syndrome. In addition, the limited evidence base for the treatment of benign SVC syndrome precludes a standardized approach to management.

We present a case of lead-associated SVC syndrome and review the complex device management decisions that were required. We also report, to our knowledge, the first published report of SVC syndrome with resultant subcutaneous edema affecting subcutaneous ICD (S-ICD) QRS screening.

## Case presentation

**Figure 1:**
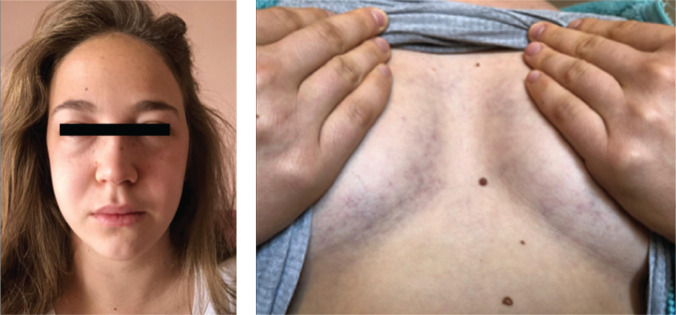
Photographs of the patient described in this case report taken at the time of presentation. She noted the development of subacute facial plethora, periorbital edema, neck fullness, inframammary venous engorgement, and inframammary ecchymosis. In certain cases of SVC syndrome, recruitment of collateral thoracic circulation can be mistaken for caput medusa, which is typically associated with liver disease.

A 22-year-old woman living in Argentina with a history of arrhythmogenic right ventricular cardiomyopathy (ARVC) due to a plakophilin-2 (*PKP-2*) mutation presented with neck fullness, facial plethora, and bilateral inframammary venous engorgement and ecchymoses **(Figure 1)**. Three years earlier, in 2017, she had received a secondary-prevention single-chamber ICD via the left axillary vein.

**Figure 2:**
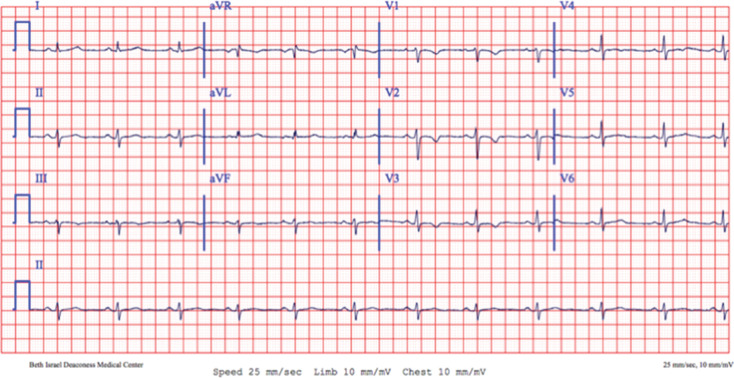
Baseline ECG captured in 2015 notable for normal sinus rhythm with T-wave inversions in the right precordial leads, consistent with the diagnosis of ARVC.

Her diagnosis of ARVC was made in 2015, at the age of 17 years, when she presented for evaluation after an episode of syncope. She had a family history of ARVC in her uncle and a cousin. Her electrocardiogram (ECG) at presentation was notable for T-wave inversions in the right precordial leads **(Figure 2)**. Cardiac magnetic resonance imaging showed no clear evidence of ARVC and an electrophysiology study failed to induce a ventricular arrhythmia. Genetic testing returned positive for a *PKP-2* mutation (W538X variant, her known familial variant). An implantable loop recorder was placed for ongoing monitoring and she was started on β-blocker therapy. In July 2017, she experienced an episode of sustained but self-terminating monomorphic ventricular tachycardia (VT) in association with exercise. Repeat cardiac magnetic resonance imaging revealed evidence of a small area of fatty replacement in the right ventricular apical region. A single-chamber ICD was implanted via the left axillary vein for secondary prevention of sudden cardiac death. She did well until October 2018 when, while playing a recreational soccer game, she experienced a sustained episode of monomorphic VT that terminated with antitachycardia pacing. Ablation was recommended but was declined by the patient. She was advised to abstain from high-intensity, long-duration exercise.

**Figure 3:**
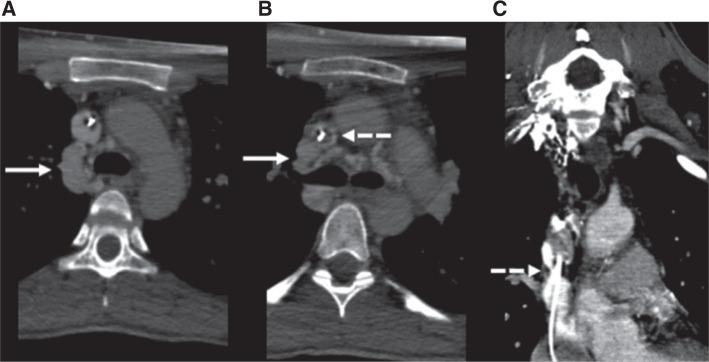
Computed tomography angiography was performed in Argentina. **A and B:** Transverse slices showing a dilated azygos vein and azygos arch (solid arrow) as well as a stenotic SVC with possible thrombus (dashed arrow). **C:** Coronal section showing the RV ICD lead coursing through a stenotic SVC (dashed arrow).

She remained symptom-free until April 2020. While performing a plank exercise, she experienced a “pop” sensation in her neck; then, during the next week, she developed bilateral inframammary ecchymoses and neck fullness. Computed tomography venography revealed stenosis at the level of the proximal to mid-SVC and a dilated azygous vein, supporting a clinical presentation most consistent with lead-associated SVC syndrome **(Figure 3)**. She was started on a direct oral anticoagulant with relatively prompt improvement in her swelling. However, three months later, despite continuous anticoagulation, her swelling recurred. At this point, she traveled from Argentina to Boston for further management.

**Figure 4:**
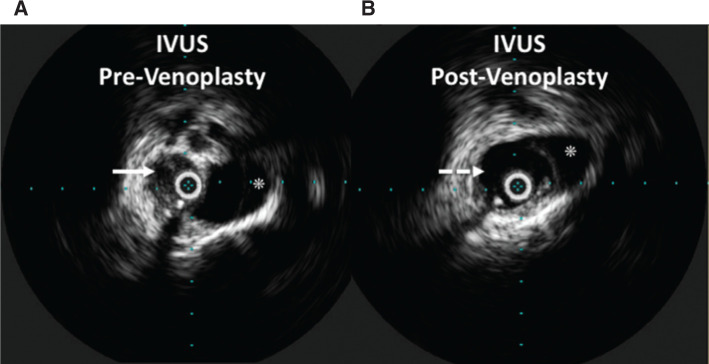
Intravascular ultrasound images of the SVC were created immediately post–lead extraction **(A)** and following serial venoplasty **(B)**. The immediate post–lead extraction image is notable for a stenotic SVC with intraluminal thrombus (solid arrow). Following three serial venoplasty balloon dilations, the SVC was noted to be expanded **(B)**. The azygos vein is visualized in both images (see asterisk).

**Figure 5:**
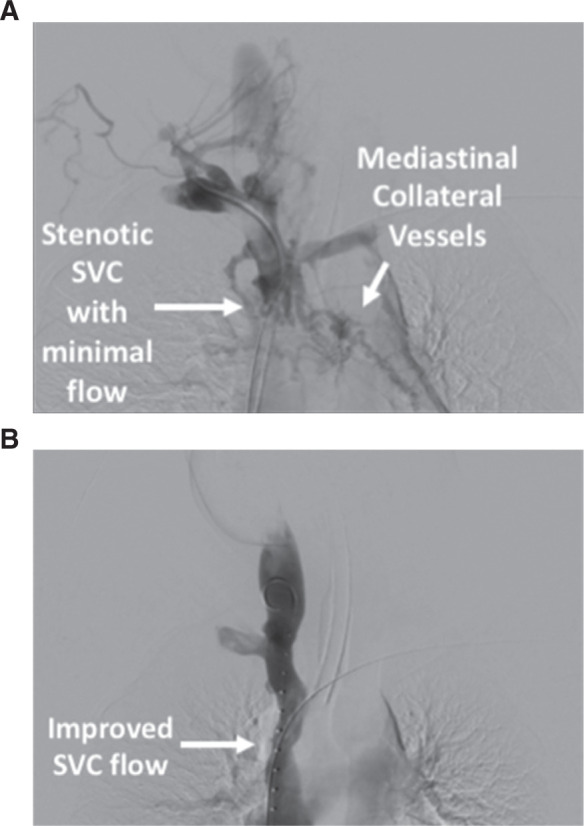
Digital subtraction angiography performed during the procedure. **A:** Post–lead extraction, prevenoplasty angiogram depicting the stenotic SVC with collateral mediastinal vessels. **B:** Post–lead extraction, postvenoplasty angiogram depicting improved SVC expansion and flow.

Laser sheath–assisted transvenous lead extraction (TLE) was performed successfully. During the same procedure, vascular surgery conducted digital subtraction venography and intravascular ultrasound imaging; both modalities revealed significant stenosis at her SVC just above the cavoatrial junction **(Figures 4 and 5)**. Serial balloon venoplasty was successful, with repeat intravascular ultrasound imaging showing significant expansion of the SVC with restoration of blood flow.

**Figure 6:**
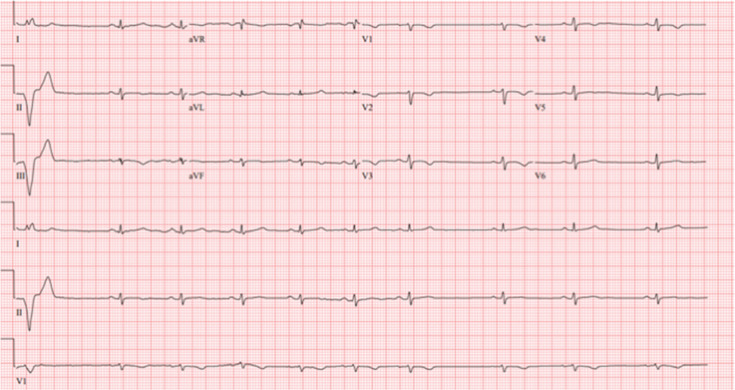
The pre–lead extraction presenting ECG demonstrating normal sinus rhythm with persistent T-wave inversions in the right precordial leads. As compared with the 2015 baseline EKG **(Figure 2)**, QRS amplitude was reduced in both the limb and precordial leads. Of note, the single premature ventricular complex seen on this 12-lead tracing has a left-bundle, left-superior axis likely originating from the right ventricle.

**Figure 7:**
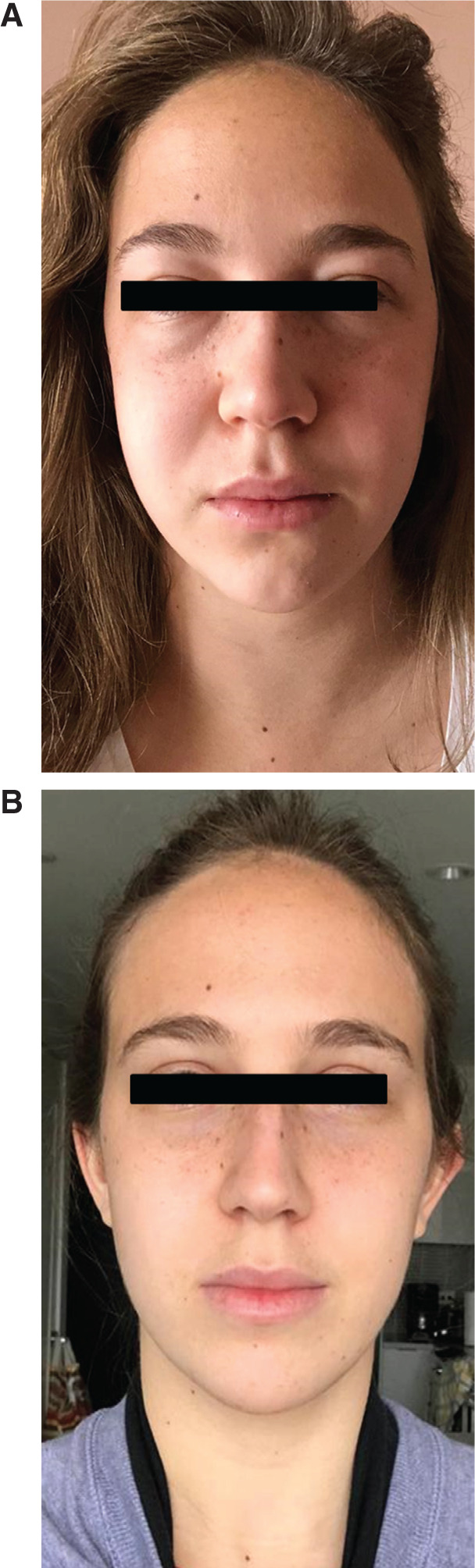
A comparison between front images of the patient taken pre–lead extraction **(A)** and two weeks post–lead extraction with venoplasty **(B)**. The post–lead extraction image shows resolution of the periorbital edema, facial plethora, and neck fullness that were previously noted.

Prior to TLE, her ECG was notable for diminished voltage throughout the limb and precordial leads as compared with her pre–SVC syndrome ECGs **(Figure 6)**. She failed S-ICD QRS vector screening despite multiple sensing vectors trialed. Following extraction, signs of venous congestion improved over the course of the next few days and were completely resolved by two weeks postprocedure **(Figure 7)**. Moreover, her QRS amplitude returned to near pre–SVC syndrome values. Repeat S-ICD QRS vector screening demonstrated several passing vectors. Repeat magnetic resonance angiography performed three weeks postprocedure demonstrated persistent patency of the SVC.

Treatment options including no ICD reimplantation, VT ablation, and S-ICD implant were discussed. The patient preferred to defer a VT ablation and chose to proceed with S-ICD implantation for secondary prevention. An S-ICD was implanted in an intermuscular location and sensing was adequate. Postprocedure anticoagulation with a direct oral anticoagulant will be maintained for at least 12 months and repeat vascular imaging is planned at six and 12 months postoperation.

## Discussion

Lead-associated SVC syndrome is a rare condition and the optimal management protocols have yet to be defined. In this specific case, there were two key management decisions that required addressing, specifically (1) what initial vascular intervention should be performed and (2) should we reimplant an ICD and, if so, how?

### Percutaneous Intervention

Lead-associated SVC syndrome carries a class I indication for TLE.^5^ However, very little has been published outside of retrospective case series with regard to how to manage residual venous obstruction. A pooled analysis of lead-associated SVC syndrome cases reviewed the literature from 1970 to 2009, identifying 74 publications with 104 total patients.^6^ Patients were stratified by the intervention received and their outcomes were evaluated. Stenting had a low recurrence rate (5%) versus venoplasty (23%); however, only 28% (7/25) of the stenting group and 10% (1/10) of the venoplasty-alone group underwent TLE prior to intervention. It is likely that lead persistence promotes restenosis, particularly in the absence of a stent, making it difficult to compare these approaches when extraction is performed first.

Other retrospective studies have evaluated venoplasty without TLE and reported a similar patency rate of 73% at two years, with an average of 2.1 procedures performed per year.^7^ Also, a more recent single-center case series described various success rates where TLE was combined with different percutaneous vascular techniques.^4^ There was significant heterogeneity with regard to the percutaneous interventions selected in this series of 16 patients. Six patients (37.5%) received an SVC stent; five of these underwent SVC balloon angioplasty prior to stent placement. Notably, no patients were managed with balloon venoplasty alone. At a median follow-up point of 5.5 years (interquartile range: 2–8.5 years), only 25% of patients experienced recurrence of symptoms post-TLE.

We believe that transvenous lead extraction is a foundational intervention in patients with lead-associated SVC syndrome. Removal of the lead should relieve the vascular insult driving venous stenosis. If a transvenous device remains necessary, stent placement may be warranted to help reduce lead-endothelial interactions that may be driving vascular remodeling. If a stent is to be placed and a transvenous system reimplanted, it is prudent for the new lead(s) to pass through the venous stent and not become “jailed” between the stent and vessel wall, which risks lead integrity and makes any future transvenous lead extractions impossible to complete.

What is also unclear is which vascular interventions should be performed post-TLE if no transvenous system is to be reimplanted. Venous stents carry significant risks, including migration, fracture, infection, pericardial tamponade, and thrombus.^8,9^ Until more is known regarding the utility of TLE plus stenting versus TLE plus venoplasty alone, we must rely on multidisciplinary case discussions with vascular surgery to create patient-specific procedural plans. In this specific case, given the young age of the patient, we were reluctant to place a stent. As such, we chose to pursue TLE with venoplasty alone with a plan to avoid reimplantation of an endovascular lead as an initial strategy.

### Subcutaneous implantable cardioverter-defibrillator screening

In regard to our patient’s ongoing rhythm management, ablation was again discussed and again declined by the patient and her family. Options for ICD therapy included epicardial or subcutaneous systems. Prior to TLE extraction, likely due to thoracic venous congestion, our patient’s QRS amplitude was diminished relative to on prior ECGs **(Figure 6)**. As such, S-ICD QRS vector screening failed to find an appropriate QRS vector that would have made her a suitable S-ICD candidate. We hypothesized that, if we were successful at relieving the SVC obstruction, her thoracic venous congestion would improve and her QRS amplitude would recover. The patient reported a marked improvement in her swelling by five days postextraction. A repeat ECG demonstrated that the QRS amplitude had returned to near-baseline values and her S-ICD QRS vector screening passed in the alternate vector both through the automatic and manual screening tools.

To our knowledge, this is the first report of a patient with lead-associated SVC syndrome who initially failed S-ICD screening but was later screened-in as a candidate following vascular intervention. The efficacy of the S-ICD depends on its ability to reliably sense the native QRS. The system uses a morphology-based sensing algorithm that analyzes the QRS signal amplitude and QRS:T-wave signal ratios to determine the appropriate sensing vector (EMBLEM S-ICD; Boston Scientific, Natick, MA, USA). Various studies have shown that the QRS sensing vector changes with frequency over time due to variations in the native QRS and T-wave amplitudes. In one retrospective study with a median follow-up time of 27.3 ± 25.3 months, 35.7% of patients experienced a change in their sensing vector during follow-up as compared with at implant.^10^ It has also been shown that QRS sensing can change with alterations in posture (eg, standing vs. supine) or with exercise.^11,12^ Eight weeks after device implant, our patient’s vectors were evaluated repeatedly while standing and briskly walking on a treadmill and we found that, in her case, the alternate vector remained stable and usable.

The screening vector for S-ICD is dynamic. This case and prior studies illustrate that a large percentage of patients have alterations in their QRS and T-wave sensing over time. It is important to consider reassessing vector sensing as part of routine clinical care for S-ICD patients.

In the case of a patient with lead-associated SVC syndrome and the need for sudden death prevention, an S-ICD is an attractive alternative. The S-ICD system mitigates the lead–vessel interaction that may be driving the pathological endothelial remodeling associated with venous obstruction. Following transvenous lead extraction, in cases where patients were not initially considered viable S-ICD candidates after failing preimplant QRS vector screening, venous congestion may improve, resulting in changes to the QRS amplitude and QRS:T-wave ratios. These patients may subsequently become S-ICD candidates following relief from SVC stenosis.

## Commentary from Dr. Zimetbaum

This case, presented by Dr. Locke as the winner of the 2020 EP Fellows Summit case competition, provides an example of the troubling issue of venous occlusion associated with transvenous pacemaker and ICD leads. Venous occlusion associated with device leads is quite common; however, data-driven guidance for the management of occlusion, including the timing and manner of intervention, is lacking. SVC obstruction is the rarest but most morbid form of venous occlusion and, once again, existing guidance for lead extraction and stent versus standalone venoplasty in this context is insufficient. Furthermore, recommendations for postextraction anticoagulation are also lacking. While we await the advent of fully leadless systems, the collection of more robust data, at least in the form of a registry, would be helpful.

This case also demonstrates the limitations of S-ICD lead sensing in its current form; chest wall edema amongst other factors such as patient posture can alter sensing vectors and influence ICD performance. Once again, with the continued refinement of these extravascular technologies, these issues should be overcome, but, in the meantime, subcutaneous edema should be considered as a limitation with the current S-ICD technology.
